# Are the Effects of Homeopathy Attributable to a Statistical Artefact? A Reanalysis of an Observational Study

**DOI:** 10.1155/2013/612890

**Published:** 2013-12-15

**Authors:** Rainer Lüdtke, Stefan N. Willich, Thomas Ostermann

**Affiliations:** ^1^Institute of Social Medicine, Epidemiology, and Health Economics, Charité University Medical Center, 10098 Berlin, Germany; ^2^Institute of Integrative Medicine, Witten/Herdecke University, Gerhard-Kienle-Weg 4, 58313 Herdecke, Germany

## Abstract

*Background*. Cohort studies have reported that patients improve considerably after individualised homeopathic treatment. However, these results may be biased by regression to the mean (RTM). *Objective*. To evaluate whether the observed changes in previous cohort studies are due to RTM and to estimate RTM adjusted effects. *Methods*. SF-36 quality-of-life (QoL) data from a German cohort of 2827 chronically diseased adults treated by a homeopath were reanalysed by Mee and Chua's modified *t*-test. *Results*. RTM adjusted effects, standardized by the respective standard deviation at baseline, were 0.12 (95% CI: 0.06–0.19, *P* < 0.001) in the mental and 0.25 (0.22–0.28, *P* < 0.001) in the physical summary score. Small-to-moderate effects were confirmed for the most individual diagnoses in physical, but not in mental component scores. Under the assumption that the true population mean equals the mean of all actually diseased patients, RTM adjusted effects were confirmed for both scores in most diagnoses. *Conclusions*. Changes in QoL after treatment by a homeopath are small but cannot be explained by RTM alone. As all analyses made conservative assumptions, true RTM adjusted effects are probably larger than presented.

## 1. Introduction

Homeopathy is a whole medical system practiced in many regions of the world [1], especially in high-income countries where it ranks as the most popular among the traditional, complementary, or alternative medicines [1–3]. In homeopathy, a diagnosis can be treated with different medicines in different patients “individualization”, depending on varying concomitant symptoms. Homeopathic medicines (remedies) are produced by alternating steps of diluting and agitating a starting substance; the resulting “potencies” quickly reach dilutions beyond Avogadro's number where the probability that one molecule of the starting substance is still present rapidly approaches zero. Such “high potencies” are often used, and their effects constitute a subject of scientific controversy [4]. Systematic reviews of placebocontrolled trials (pooling a great variety of diseases and ailments) [5–7] have shown inconsistent results.

Treatment by homeopaths is typically regarded as a complex health intervention, which cannot be reduced to the efficacy of homeopathic medicines alone. It is claimed that the special style of case-taking, a different approach on how to manage conventional therapies, and specific life-style recommendations must both be considered as intrinsic parts of a homeopathic treatment. If so, randomised controlled trials on homeopathic medicines alone might be inappropriate or at least insufficient to research homeopathy as a whole [8, 9]. On this background large-scaled, uncontrolled cohort studies have been conducted which aim to assess health effects by global, patient-centered outcome measures (outcome studies). These studies consistently reported that quality of life improves and/or the severity of health complaints decreases in the majority of patients after treatment by a homeopath (see, e.g., [10–12]).

This is not an astonishing result, because patients usually seek treatment when their health is worse than average. Thus, an alleviation of their illnesses can easily be mistaken for an effect from the initiation of treatment, although it only represents natural variability. This phenomenon is widely known as “regression to the mean” (RTM), first described by Galton [13]. In a more general context RTM occurs in situations of repeated measurements when extremely large or small values are followed by measurements in the same subjects that on average are closer to the mean of the basic population. Such changes are likely to be interpreted as a real drift, although they just might be artificially coming from the fact that the sampling of values was not random but selected from the extremes. Thus, RTM is a statistical artefact and should be conceptually distinguished from treatment. Moreover, as the constituting mechanisms are completely different, it also cannot be considered as part of a placebo effect.

In a real data situation, however, separating RTM from treatment effects might be difficult, because the process how patients are selected into a study is not known or cannot formally be described (there are exceptions, e.g., when all patients are included whose pain values exceed a defined threshold).

Twenty years ago Mee and Chua [14] have proposed a statistical procedure which allows to estimate a “treatment effect” taking into account that RTM might be present. In this paper we adopted Mee and Chua's procedure (and its recently published graphical extension [15]) to estimate whether the observed changes in homeopathic outcome studies are due to RTM. Special interest is laid on the outcome study by Witt et al. which found that health related quality of life (QoL) in chronically diseased patients improved substantially after starting treatment by a homeopath [10].

## 2. Methods

### 2.1. The Study

This was a reanalysis of a formerly published outcome study which evaluated the course of disease under a treatment by a homeopath for a wide range of diagnoses under the conditions of usual care in Germany [8]. Each patient (children and adults) who attended one of 104 participating homeopathic physicians in Germany or Switzerland was followed up for two years with measurements of health status taken at baseline (first contact to a homeopathic physician for the actual complaints), and 3, 12, and 24 months after treatment started. All physicians followed an individualised, classical approach on homeopathy.

### 2.2. Outcomes

In this paper, we restricted our analysis to the 12 months results of the SF-36 questionnaire which measures QoL in eight scales and two summary scores (mental component MCS and physical component PCS). All scales and summary scores were transformed to American norm-based scores, such that the American norm population has an average score of 50 and a standard deviation of 10 [16].

### 2.3. Patients

Patient subgroups were built on ICD-9 diagnoses made by the homeopathic physicians at baseline. Here, we selected a priori the most frequent diagnoses in adults (see Table  1 in [17]). Although not ranking top we added asthma and psoriasis because here SF-36 population based norm values were available. As multiple diagnoses were allowed, one patient could be part of several diagnoses subgroups.

Diagnosis specific analyses were defined a secondary in this paper and the respective statistical tests were only performed for descriptive reasons. Consequently in this study all *P* values should not be interpreted as error probabilities.

### 2.4. Statistics

For each subgroup we applied Mee and Chua's procedure [14] to test whether the observed change in outcome might be due to RTM and to estimate “RTM adjusted effects of homeopathy.” This term does not mean “specific and causal effect of homeopathic treatment” but is related to any change of health status due to the fact that the patient started being treated by a homeopath. This definition excludes RTM but includes nonspecific effects (placeboeffects) or effects of concomitant health interventions to be responsible for this change.

Mee and Chua's modified *t*-test requires the true mean in the target population to be known, a requirement that seldom can be fulfilled. We therefore took the SF-36 mean summary scores from the 1998 German health survey as proxies. The German health survey provided data for the general population, which includes diseased and healthy persons (MCS: 51.54, PCS: 50.21), for the subset of all actually diseased patients (MCS: 48.89, PCS: 47.16), and for chronically ill patients with low back pain (PCS: 44.79, MCS: 48.25), skin diseases (PCS: 47.95, MCS: 43.74), hypertension (PCS: 44.38, MCS: 48.72), or allergies (PCS: 50.00, MCS: 48.44) [16].

Inserting these values, however, might be a conservative approach (meaning that the RTM effects are overestimated and the treatment effects are underestimated), especially when the means were taken from the general population. According to suggestions of Ostermann et al. [15] we therefore varied this mean systematically over a range of reasonable values, ran the Mee-Chua algorithm for each mean separately, and plotted the RTM adjusted effects and confidence intervals (CI) against this mean. This should give an overall impression about how RTM affected the data.

All estimates were presented as standardized mean changes, dividing the RTM adjusted effect by the respective standard deviation at baseline. They were classified as large if >0.8, as medium if >0.5, as small if >0.2, and as very small if <0.2.

All descriptive statistics are given as mean ± standard deviation. Reported CIs are at a 95% level.

## 3. Results

In total 3981 patients were included in the study, of these 2827 were adults (70% women, 39.83 ± 12.3 years, 30% men, 42.3 ± 13.0 years) who contributed data to the SF-36 physical and mental summary scores. The majority of these patients suffered from chronic diseases, with all types of headache (*N* = 228), eczemas (*N* = 211), allergies (*N* = 165), and depression (*N* = 164) ranking top. QoL was predominantly limited on the MCS and to a lower degree on the PCS (Table 1). Improvements were more pronounced in the MCS compared to the PCS. In a standardised scale (ratio of mean change divided by the standard deviation at baseline) MCS score changes were moderate to large and ranged from 0.25 in hypertension to 0.91 in depression. PCS scores were small to medium and ranged from 0.13 in sleep disturbances to 0.51 in recurrent infects.

**Figure 1:**
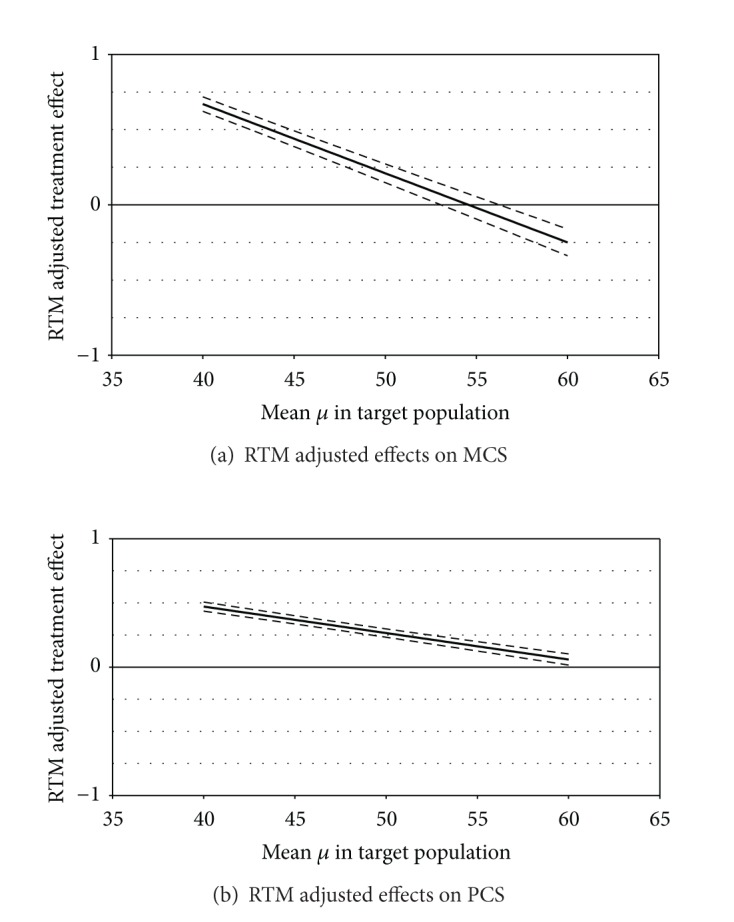
RTM adjusted effects on (a) MCS and (b) PCS under various scenarios for the mean in the target population (*x*-axes show possible values for *μ* in a reasonable range, and the *y*-axes show the respective RTM adjusted effects; thick line: estimated effect, thin lines: 95% confidence intervals).

Figure 1 shows that RTM adjusted effects can be confirmed on MCS and PCS in the whole study population for a wide range of assumptions on the unknown population mean *μ*. There is a statistically significant RTM adjusted effect if *μ* is smaller than 61.7 in PCS or smaller than 53.3 in MCS. In detail, inserting the mean scores of the general German population into Mee-Chua's procedure yielded small but statistically significant RTM adjusted effects on MCS (0.12, CI: 0.06 to 0.19, *P* = 0.0003; Table 2) and PCS (0.25, CI: 0.22 to 0.28, *P* < 0.0001; Table 3).

**Figure 2:**
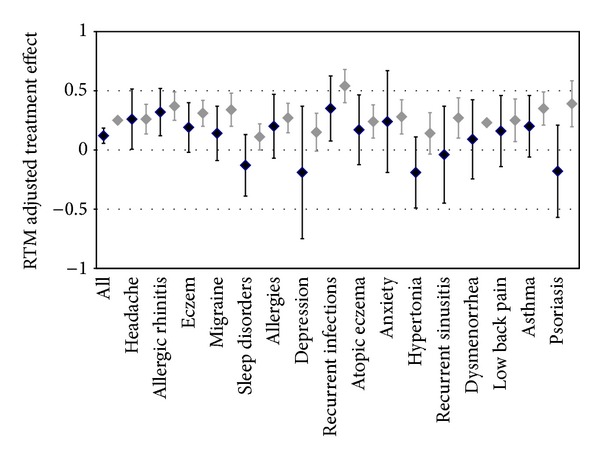
RTM adjusted effects, assuming the patient's true mental (grey lines) and physical (black lines) quality of life to be identical to the general population (MCS: 51.54, PCS: 50.21).

Generally these effects were smaller on the MCS than on the PCS (Figure 2). Statistical confirmation of effects failed in most disease subgroups in MCS, with the exception being recurrent infects. In contrast, significant effects were found for several diseases on the PCS, including migraine, allergies, anxiety disorders, and asthma. The largest effects were found in migraine, psoriasis, and asthma.

The situation changes, if the mean scores of the actually diseased German population were inserted into Mee-Chua's procedure. Again RTM adjusted effects in MCS were generally smaller than in PCS, but now these effects could be confirmed by statistical means in migraine, headache, eczemas, allergies, recurrent infects, atopic eczema, and asthma (Table 2). There are, however, some few exceptions where treatment effects could not be found. In recurrent sinusitis, for example, we found no effect in PCS and a moderate but statistically not significant effect in the MCS.

The results could be corroborated if disease specific mean scores were inserted instead (Tables 2 and 3). RTM adjusted effects on MCS and PCS were small or moderate and statistically significant in most cases.

**Figure 3:**
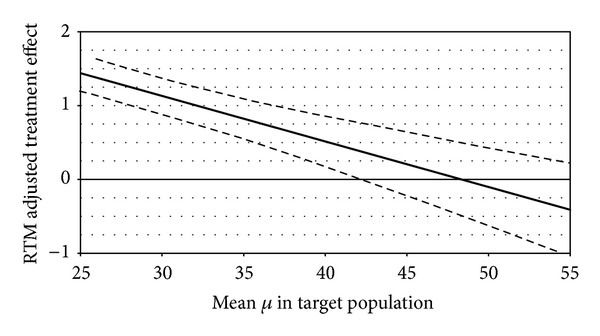
RTM adjusted effects on MCS in depression under various scenarios for the mean in the target population (*x*-axes show possible values for *μ* in a reasonable range, and the *y*-axes show the respective RTM adjusted effects; thick line: estimated effect, thin lines: 95% confidence intervals).

Although the patients improved considerably in MCS from 29.2 ± 10.5 to 38.8 ± 13.5, we were not able to confirm an effect in patients with depression. If we abstained from the precondition that depressive patients do have the same QoL as the general German population and instead assume the mental QoL to be severely limited in the target population, we would have found significant effects of medium size (Figure 3). A mean MCS of 42.0 in the target population, for example, yields a RTM adjusted effect of 0.38 (CI: 0.01 to 0.76), a mean of 40.0 leads to an RTM adjusted effect of 0.51 (CI: 0.17 to 0.85).

## 4. Discussion 

In this paper we reanalysed data from a previously published cohort study, which evaluated the changes in health effects in patients that received treatment by a homeopath in a usual care situation. In a cohort study without control group these changes are composed of several factors, including specific effects of the homeopathic treatment or other concomitant interventions, placeboeffects, and regression to the mean, a statistical artefact that mocks a treatment effect because of selected, nonrandom sampling from the extremes. With this reanalysis we were able to demonstrate in various scenarios that the reported improvements after treatment by a homeopath cannot completely be attributed to RTM. RTM adjusted effects (summarising specific and nonspecific effects) were small or moderate but different from zero.

To our knowledge this is the first attempt to separate RTM effects from pure “treatment effects” in homeopathy and to estimate health changes that are not affected by statistical artefacts. Our results provide an overall impression of what can be expected when a chronically diseased patient starts a treatment by a homeopath. Expectations, however, should not be too exaggerated. An effect size of 0.25, as estimated for the PCS in our study, is small, because the estimated change in quality of life is only one quarter of the population's standard deviation. If one sets this standard deviation to be 10 points (as it is approximately true in the general population [10]), this translates into only 2.5 PCS score points.

It is difficult to compare these results to others. Although RTM adjusted effects have been calculated in various studies, this was never done with quality-of-life outcomes. Rough calculations from the literature (hereby assuming the pre- postcorrelations to be 0.7), however, suggest that RTM adjusted effects were similar in studies on acupuncture given additionally to routine care in patients suffering from chronic low back pain (MCS: 0.20, PCS: 0.22 [18]), osteoarthritis (MCS: 0.08, PCS: 0.22 [19]), or chronic headache (MCS: 0.12, PCS: 0.15 [20]). Similarly, Angst et al. found a multimodal inpatient rehabilitation program to have an (not RTM adjusted) effect on the PCS of 0.30 in patients with hip or knee osteoarthritis [21]. From this they determined the minimally clinical important difference at 2 score points, corresponding to an effect of 0.26.

According to the above said it was not the homeopathic medicines but the overall homeopathic approach, which observed here. This also included extensive time for case-taking, specific life-style advice, and the prescription of conventional, nonhomeopathic drugs whenever the homeopathic physician acts as he/she sees fit. Indeed during the study period half of the patients (50.3%) noted additional visits to nonhomeopathic doctors (gynaecologists and dentists excluded) and one fourth (26.8%) received conventional drugs [10]. RTM adjusted effects, however, did not considerably differ between patients who received additional conventional treatment (PCS: 0.27, MCS: 0.12) and those who did not (PCS: 0.24, MCS: 0.12). In this sense homeopathy and conventional medicine must not be seen as mutually exclusive, but complementary.

RTM is ubiquitous and not limited to medical or biologic aspects. It can be formally described by the distribution of data and the process of selection, that is, which measurements are taken at first and how it is decided to repeat them. Although the notion of RTM has a history of more than 100 years, the number of mathematical solutions is limited. Most of them deal with the situation that the first measurement is taken at random, and a second measurement is made, if and only if the value of the first exceeds a defined threshold [22–24]. There are, however, samples that do not fall into this scheme. One of them is the cohort study we reanalyzed in this paper. Here each patient was included who presented for the first time at a homeopathic physician and quality of life was repeatedly recorded for all included patients. There was clearly no threshold defining who received the homeopathic treatment and who did not. To our knowledge only two approaches to this problem exist. One of them, Hannan's stochastic censoring approach, is not applicable for this study, because it also needs data from those patients who were not subjected for a second measurement [25]. Mee and Chua's modified *t*-test is independent from the selection process but relies on the rather restrictive assumption, that the mean of the target population is known. As this is seldom the case the resulting estimate for the RTM adjusted effect can only be seen as a proxy for the true effect. In this paper we tried to overcome this limitation by inserting various plausible values in a sensitivity analysis. This, however, can only give an overall impression but cannot give a definite answer on the size of RTM adjusted effects.

Our data showed that patients improved better in mental than in physical aspects. RTM adjusted effects, however, were larger in the PCS than in the MCS. This is not a contradiction but can be explained from the concept of RTM. In our study patients were primarily affected in mental aspects, and the respective scores were considerably lower than their physical counterparts. Sampling was more from the extremes, so it was more likely that RTM affected mental scores more than physical scores. In consequence more parts of the overall effect are attributed to RTM but not to the true effect.

RTM adjusted effects on the MCS were especially small in depression, sleep disorders, and hypertension (and were even negative in some scenarios). For psoriasis and sleep disorders probably the same argument holds as in depression (see Section 3). These patients are often severely limited in mental and social aspects of quality of life. Comparing them to the general diseased population might be a too conservative approach. Indeed, if one inserts the psoriasis disease specific MCS score of 43.74 the RTM adjusted effect approaches significance. A somewhat different argument holds for hypertension. It appeared that the hypertensive patients in our study were much more limited in their mental quality of life than the hypertensive patients of the general German population. Obviously our patients were even a subsample of all hypertensive patients, having multiple comorbidities and seeing the need for being helped by a physician.

This paper reports numerous *P* values. Thus, a multiple statistical error is likely, if each *P* value is interpreted as an error probability to test a specific hypothesis. As already mentioned in Section 2 in this study *P* values should be interpreted descriptively instead. It was primarily the size of the effect estimates and the pattern of *P* values that mattered. Anyhow, the main outcome would have been statistically significant, if one had adjusted the *P* values, for example, by the Bonferoni-Holm procedure.

The question of RTM-attributed effects should be clearly distinguished from the question whether homeopathy has any treatment effect beyond placebo. This is still a matter of debate [26]. In 2005 Shang et al. reviewed 110 randomized placebocontrolled trials on homeopathy [5] and found that smaller trials were more likely to produce results in favour of homeopathy than larger trials and that no overall effect of homeopathic medicines can be found that exceeds a placebo effect (odds ratio 0.88; CI: 0.65 to 1.19). More recent systematic reviews make a clear distinction between the effects of (highly diluted) homeopathic medicines and the effects of a homeopathic package of care [9].

Only few randomized controlled trials exist which compare a homeopathic package of care with a conventional treatment. Most evidence comes from uncontrolled cohort studies where a large number of patients was treated by a homeopath and systematically followed up for a defined time period. A complex and refined analysis of these data (beyond simply calculating the mean change of scores) is urgently needed; this paper should be regarded as a first step in this direction.

This view is compatible with the concept of comparative effectiveness research, which aims for valid decision making in usual care [27]. In this context researchers are explicitly encouraged to analyse respective data (e.g., from registries and cohort studies) as a basis for forthcoming controlled trials [28]. Our data should help to design future trials comparing a homeopathic with a conventional approach.

## 5. Conclusions

In our paper we showed that the effects on quality of life observed in patients receiving homeopathic care in a usual care setting are small or moderate at maximum, but cannot be explained by RTM alone. Due to the uncontrolled study design they may, however, completely be due to nonspecific effects. All our analyses made a restrictive and conservative assumption, so the true treatment effects might be larger than shown.

## Figures and Tables

**Table 1:** Baseline values and changes after 12 months of SF-36 summary scores (mean ± standard deviation).

Disease (ICD-9)	*N*	Mental component MCS			Physical component PCS		
		Baseline	Change	Standardised change*	Baseline	Change	Standardised change*
All	2827	39.2 ± 13.4	5.6 ± 11.8	0.42 ± 0.88	46.9 ± 14.0	3.2 ± 9.3	0.23 ± 0.66
Headache (346.9 or 784.0)	228	38.8 ± 13.3	5.6 ± 11.7	0.42 ± 0.88	45.8 ± 13.3	3.8 ± 9.9	0.29 ± 0.74
Allergic rhinitis (477.9)	227	41.6 ± 14.0	6.3 ± 11.0	0.45 ± 0.79	49.7 ± 13.6	3.9 ± 10.0	0.29 ± 0.74
Eczema (692.9)	211	41.6 ± 13.7	6.1 ± 11.9	0.45 ± 0.87	52.0 ± 12.7	2.7 ± 9.0	0.21 ± 0.71
Migraine (349.6)	210	41.1 ± 13.1	5.1 ± 11.9	0.39 ± 0.91	45.3 ± 12.1	4.8 ± 10.3	0.40 ± 0.85
Sleep disturbances (780.5)	204	32.6 ± 11.6	6.4 ± 12.7	0.55 ± 1.09	44.0 ± 13.1	1.7 ± 8.3	0.13 ± 0.63
Allergy (995.3)	165	41.1 ± 13.5	6.0 ± 12.5	0.44 ± 0.93	50.6 ± 13.9	2.7 ± 8.8	0.19 ± 0.63
Depression (311.0)	164	29.2 ± 10.5	9.6 ± 13.3	0.91 ± 1.27	46.6 ± 13.9	2.5 ± 10.6	0.18 ± 0.76
Recurrent infections (796.9)	153	39.9 ± 13.1	7.7 ± 11.8	0.59 ± 0.90	47.8 ± 12.0	6.1 ± 9.2	0.51 ± 0.77
Atopic eczema (691.8)	145	39.9 ± 13.0	6.0 ± 12.1	0.46 ± 0.93	51.4 ± 12.5	2.1 ± 9.4	0.17 ± 0.75
Anxiety disorder (300.0)	142	34.4 ± 12.1	9.9 ± 13.2	0.82 ± 1.09	49.9 ± 13.2	2.9 ± 9.3	0.22 ± 0.70
Hypertension (401.9)	137	35.4 ± 13.3	3.3 ± 9.8	0.25 ± 0.74	38.0 ± 14.1	3.7 ± 8.4	0.26 ± 0.60
Recurrent sinusitis (473.9)	130	38.0 ± 10.6	5.9 ± 12.5	0.56 ± 1.18	46.7 ± 12.8	3.9 ± 10.3	0.30 ± 0.80
Dysmenorrhea (625.3)	128	38.8 ± 13.4	6.2 ± 12.6	0.46 ± 0.94	49.5 ± 12.8	2.6 ± 8.9	0.20 ± 0.70
Low back pain (724.2)	128	39.8 ± 12.2	5.3 ± 10.7	0.43 ± 0.88	43.3 ± 14.5	3.2 ± 9.3	0.22 ± 0.64
Asthma (493.9)	118	41.2 ± 14.0	5.8 ± 10.7	0.41 ± 0.76	47.8 ± 13.3	3.8 ± 7.5	0.29 ± 0.56
Psoriasis (696.1)	81	40.5 ± 13.6	3.7 ± 12.8	0.27 ± 0.94	47.4 ± 15.2	4.8 ± 9.6	0.32 ± 0.63

*Change divided by standard deviation at baseline.

**Table 2:** RTM adjusted effects after 12 months on MCS for various scenarios.

Sampling from …	… the general population*		… the actually diseased population*		… specifically diseased patients*	
Disease (ICD-9)	Effect (95% CI)	*P*	Effect (95% CI)	*P*	Effect (95% CI)	*P*
All	0.12 (0.06 to 0.19)	0.0003	0.18 (0.14 to 0.23)	<0.0001		
Headache (346.9 or 784.0)	0.26 (0.00 to 0.51)	0.0470	0.26 (0.09 to 0.44)	0.0037		
Allergic rhinitis (477.9)	0.32 (0.12 to 0.51)	0.0014	0.33 (0.19 to 0.47)	<0.0001	0.35 (0.21 to 0.48)	<0.0001
Eczemas (692.9)	0.19 (−0.02 to 0.4)	0.0688	0.26 (0.11 to 0.40)	0.0007		
Migraine (349.6)	0.14 (−0.09 to 0.37)	0.2416	0.19 (0.03 to 0.36)	0.0178		
Sleep disturbances (780.5)	−0.33 (−0.70 to 0.05)	0.0904	−0.13 (−0.39 to 0.13)	0.3120		
Allergies (995.3)	0.20 (−0.07 to 0.47)	0.1368	0.25 (0.07 to 0.44)	0.0082	0.27 (0.09 to 0.46)	0.0042
Depression (311.0)	−0.19 (−0.75 to 0.37)	0.4989	−0.03 (−0.41 to 0.36)	0.8938		
Recurrent infections (796.9)	0.35 (0.07 to 0.62)	0.0139	0.36 (0.17 to 0.55)	0.0002		
Atopic eczema (691.8)	0.17 (–0.13 to 0.46)	0.2640	0.22 (0.02 to 0.43)	0.0323	0.36 (0.21 to 0.52)	<0.0001
Anxiety (300.0)	0.24 (−0.19 to 0.67)	0.2707	0.29 (0.00 to 0.59)	0.0518		
Hypertension (401.9)	−0.19 (−0.49 to 0.11)	0.2200	−0.07 (−0.28 to 0.14)	0.4978	−0.07 (−0.27 to 0.14)	0.5242
Recurrent sinusitis (473.9)	−0.04 (−0.45 to 0.37)	0.8514	0.08 (−0.19 to 0.35)	0.5621		
Dysmenorrhea (625.3)	0.09 (−0.25 to 0.42)	0.6175	0.17 (−0.07 to 0.40)	0.1589		
Low back pain (724.2)	0.16 (−0.14 to 0.46)	0.2914	0.20 (0.00 to 0.41)	0.0532	0.22 (0.02 to 0.42)	0.0003
Asthma (493.9)	0.20 (−0.06 to 0.46)	0.1231	0.25 (0.07 to 0.43)	0.0075	0.28 (0.13 to 0.42)	0.0003
Psoriasis (696.1)	−0.18 (−0.57 to 0.21)	0.3557	−0.03 (−0.30 to 0.24)	0.8475	0.17 (−0.03 to 0.38)	0.0961

*As defined by the German health survey, which shows data for the general population, including diseased and healthy persons, for the subset of all actually diseased patients and for chronically ill patients suffering from specific diseases [14]; see Section 2.4.

**Table 3:** RTM adjusted effects after 12 months on PCS for various scenarios.

Sampling from …	… the general population*		… the actually diseased population*		… specifically diseased patients*	
Disease (ICD-9)	Effect (95% CI)	*P*	Effect (95% CI)	*P*	Effect (95% CI)	*P*
All	0.25 (0.22 to 0.28)	<0.0001	0.30 (0.27 to 0.33)	<0.0001		
Headache (346.9 or 784.0)	0.26 (0.13 to 0.38)	0.0001	0.33 (0.21 to 0.44)	<0.0001		
Allergic rhinitis (477.9)	0.37 (0.25 to 0.49)	<0.0001	0.45 (0.33 to 0.56)	<0.0001	0.36 (0.25 to 0.48)	<0.0001
Eczemas (692.9)	0.31 (0.20 to 0.42)	<0.0001	0.38 (0.26 to 0.50)	<0.0001		
Migraine (349.6)	0.34 (0.20 to 0.48)	<0.0001	0.40 (0.28 to 0.53)	<0.0001		
Sleep disturbances (780.5)	0.05 (−0.07 to 0.17)	0.3714	0.11 (0.00 to 0.22)	0.0498		
Allergies (995.3)	0.27 (0.14 to 0.39)	<0.0001	0.33 (0.20 to 0.45)	<0.0001	0.26 (0.14 to 0.38)	<0.0001
Depression (311.0)	0.15 (−0.01 to 0.31)	0.0600	0.22 (0.07 to 0.37)	0.0036		
Recurrent infections (796.9)	0.54 (0.40 to 0.68)	<0.0001	0.60 (0.46 to 0.73)	<0.0001		
Atopic eczema (691.8)	0.24 (0.10 to 0.38)	0.0011	0.31 (0.17 to 0.46)	<0.0001	0.28 (0.14 to 0.41)	<0.0001
Anxiety (300.0)	0.28 (0.13 to 0.42)	0.0002	0.34 (0.20 to 0.49)	<0.0001		
Hypertension (401.9)	0.14 (−0.03 to 0.32)	0.1134	0.19 (0.04 to 0.35)	0.0154	0.24 (0.10 to 0.38)	0.0011
Recurrent sinusitis (473.9)	0.27 (0.10 to 0.44)	0.0018	0.36 (0.20 to 0.51)	<0.0001		
Dysmenorrhea (625.3)	0.23 (0.09 to 0.37)	0.0015	0.31 (0.17 to 0.44)	<0.0001		
Low back pain (724.2)	0.25 (0.07 to 0.43)	0.0070	0.27 (0.11 to 0.43)	0.0014	0.29 (0.13 to 0.45)	0.0004
Asthma (493.9)	0.35 (0.21 to 0.49)	<0.0001	0.36 (0.23 to 0.49)	<0.0001	0.36 (0.23 to 0.49)	<0.0001
Psoriasis (696.1)	0.39 (0.2 to 0.59)	<0.0001	0.46 (0.27 to 0.64)	<0.0001	0.42 (0.24 to 0.59)	<0.0001

*As defined by the German health survey, which shows data for the general population, including diseased and healthy persons, for the subset of all actually diseased patients and for chronically ill patients suffering from specific diseases [14]; see Section 2.4.
